# Supplementary material for: Splice-Junction-Based Mapping of Alternative Isoforms in the Human Proteome
Source: Cell Rep. Author manuscript; Available in PMC 2020 Jan 15. (PMC6961840; doi:10.1016/j.celrep.2019.11.026)
Supplement: 3 [file NIHMS1546469-supplement-3.zip › DF2/PXD0010154/Spleen-27-Q9NR56-AAQYQVNQAAAAQAAATAAAMGIPQAVLPPLPK.pdf]

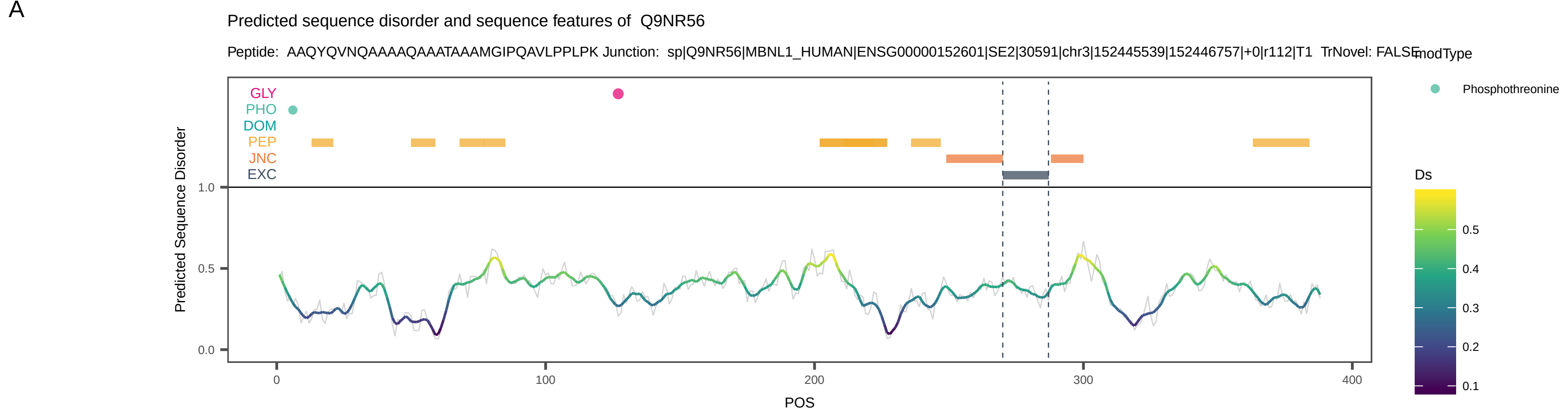
A
Predicted sequence disorder and sequence features of Q9NR56
Peptide: AAQYQVNQAAAAQAAATAAAMGIPQAVLPPLPK Junction: sp|Q9NR56|MBNL1_HUMAN|ENSG00000152601|SE2|30591|chr3|152445539|152446757|+0|r112|T1 TrNovel: FALSE
modType
Phosphothreonine
GLY
PHO
DOM
PEP
JNC
EXC
Predicted Sequence Disorder
1.0
0.5
0.0
0
100
200
300
400
POS
Ds
0.5
0.4
0.3
0.2
0.1

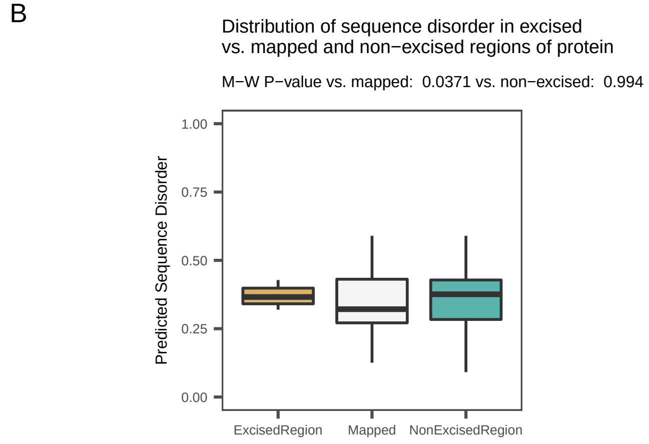
B
Distribution of sequence disorder in excised
vs. mapped and non−excised regions of protein
M−W P−value vs. mapped: 0.0371 vs. non−excised: 0.994
Predicted Sequence Disorder
1.00
0.75
0.50
0.25
0.00
ExcisedRegion
Mapped
NonExcisedRegion

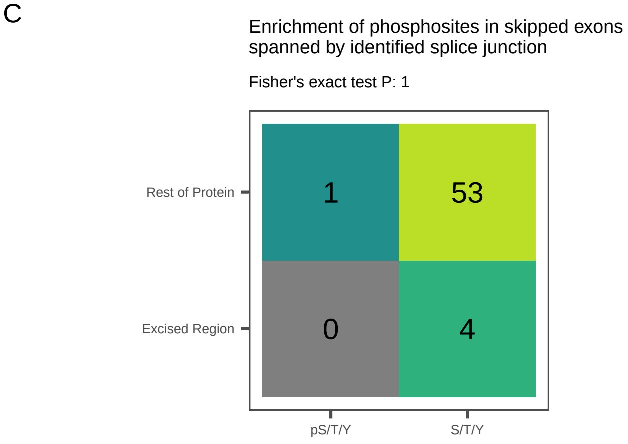
C
Enrichment of phosphosites in skipped exons
spanned by identified splice junction
Fisher's exact test P: 1
Rest of Protein
Excised Region
1
53
0
4
pS/T/Y
S/T/Y
